# A high-throughput DNA FISH protocol to visualize genome regions in human cells

**DOI:** 10.1016/j.xpro.2021.100741

**Published:** 2021-08-16

**Authors:** Elizabeth H. Finn, Tom Misteli

**Affiliations:** 1 Center for Cancer Research, National Cancer Institute, National Institutes of Health, Bethesda, MA, 20892, USA

**Keywords:** Cell Biology, Single Cell, Cell-based Assays, High Throughput Screening, Microscopy, Molecular Biology, In Situ Hybridization

## Abstract

Here, we describe an end-to-end high-throughput imaging protocol to visualize genomic loci in cells at high throughput using DNA fluorescence *in situ* hybridization, automated microscopy, and computational analysis. This is particularly useful for quantifying patterns of heterogeneity in relative gene positioning or differences within subpopulations of cells. We focus on important experimental design and execution steps in this one-week protocol, suggest ways to ensure and verify data quality, and provide practical solutions to common problems.

For complete details on the generation and use of this protocol, please refer to Finn et al. (2019).

## Highlights

• DNA fluorescence *in situ* hybridization technique for high-content imaging
• Optimized for use in a 384-well plate format with cultured cells
• One-week protocol to assess thousands of cells per condition in tens of conditions
• Thorough discussion of troubleshooting for new probes, probe types, and cell types

## Graphical abstract

**Figure:**
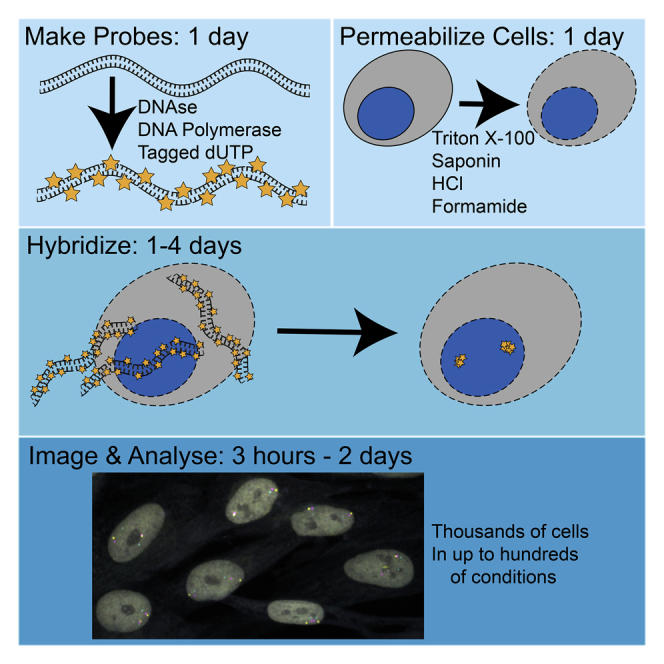

## Teaser

Here, we describe an end-to-end high-throughput imaging protocol to visualize genomic loci in cells at high throughput using DNA fluorescence *in situ* hybridization, automated microscopy, and computational analysis. This is particularly useful for quantifying patterns of heterogeneity in relative gene positioning or differences within subpopulations of cells. We focus on important experimental design and execution steps of this one-week protocol, suggest ways to ensure and verify data quality, and provide practical solutions to common problems.

## Before you begin

The protocol below describes the specific steps for using nick translated FISH probes generated from Bacterial Artificial Chromosomes (BACs) to visualize genomic regions in skin fibroblasts. However, we have used this protocol with minor adjustments for many probe types (nick translated probes that are directly labeled or labeled with a hapten such as biotin, probes generated from Bacterial Artificial Chromosomes, or fosmids, or plasmids, and oligo-library based probes; troubleshooting 1) and in numerous cell types (other adherent cells such as HCT116, HBEC, and PANC-1; pluripotent stem cells such as H1 hESCs and induced pluripotent stem cells; and suspension-grown cells such as Jurkat; troubleshooting 2).

### Grow cells on 384-well plates

**Timing: 2 days to 1 week, depending on cell doubling time and treatment conditions**

1. Dissociate cells by treating with trypsin for 5 min

***Alternatives:*** Any cell dissociation reagent can work well at this step.

2. Neutralize with equal volume trypsin-neutralizing solution or cell culture media
3. Lift by pipetting in a serological pipette several times
4. Spin 5 min at 250 *g* to pellet
5. Meanwhile, count cells
6. Decant residual media, resuspend in fresh media to get approximately 140,000 cells/mL

**Figure 1:**
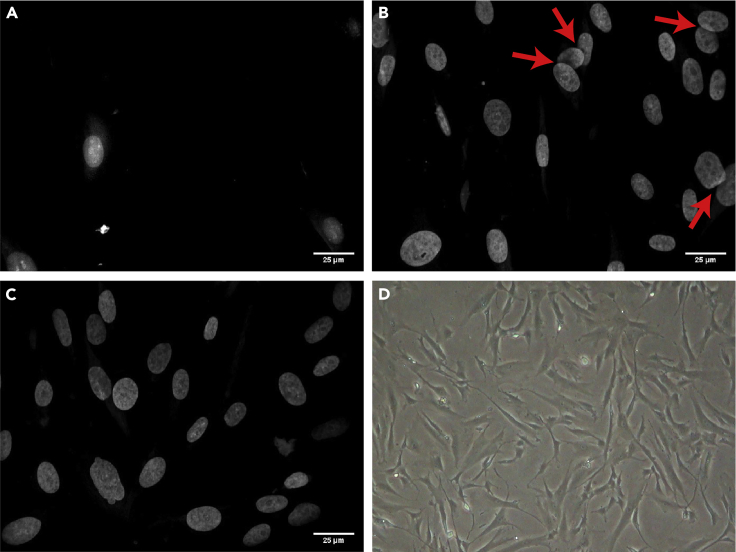
DAPI staining to measure appropriate cell density (A) Sparse cells are likely stressed and will require additional imaging time to sample fully. (B) Dense cells often overlap, confounding automated segmentation methods at overlaps (red arrows). (C) Ideally plated cells at approximately 80% confluence. (D) 80% confluent cells imaged at 4× in a dissecting scope. Scale bars: 25 microns.

***Note:*** This assumes an optimal seeding density of ∼7,000 cells per well. Depending on subsequent steps, culture time, and cell type, you will likely need to alter the plating density at this step. We recommend doing a preliminary experiment testing multiple seeding densities to determine optimal plating density, which is approximately 80% confluence at the time of imaging. (Figure 1)

7. Add 50 μL cell suspension to each well
8. Spin 30 s to remove bubbles

***Optional:*** Rest plate at 20°C–22°C for 30 minutes. This step slows adhesion and can be useful if your cells tend to be clumpy. (Troubleshooting 3)

9. Grow at least 24 h at 37°C

### Determination of optimal cell plating density

**Timing: 2 days, mostly inactive**

***Note:*** We recommend performing a cell titration as a first experiment to see how your cells grow on 384-well plates, how densely you should plate them, and whether you need to consider additional steps like gentle medium exchanges with a sponge from the outset (for more information about proper imaging density, see troubleshooting 2 and Figure 1; for more information about when gentle exchanges may be necessary see troubleshooting 3).

10. Dissociate, spin, and count cells as above (steps 1–5), but plate cells in a density gradient between 1,000 and 20,000 cells per well, with three wells per condition.
11. After 24 h growth, fix cells by adding 50 μL 8% PFA in PBS for a final concentration of 4% PFA.
12. Incubate 15 min at 20°C–22°C.
13. Rinse three times in PBS.
14. Incubate with DAPI diluted in PBS for 10 min at 20°C–22°C.
15. Image and determine maximum density before cells begin overlapping.

### Preparation of buffers, DNA, and reagents

**Timing: 4 days**

***Note:*** We recommend a multi-step procedure to grow sufficient quantities of bacteria from which to purify BACs as they are low-copy plasmids. The yield and purity of the starting DNA is a strong determinant of the efficiency of nick translation. BACs are generally shipped as agar stabs. Most BACs are chloramphenicol-resistant and we grow them in LB with freshly-added chloramphenicol to a final concentration of 25μg/mL, but double check when ordering new BAC clones.

16. Grow bacteria from single colony into large-scale culture
  a. Streak bacteria onto LB agar plate with appropriate selective antibiotic, grow 18–24 h
  b. Pick colonies and grow 4 mL cultures in LB + appropriate antibiotic 18–24 h
    ***Optional:*** Clones should be kept as a glycerol stock. We recommend making glycerol stocks at this stage when prepping from agar stabs.
  c. Use 4 mL culture to inoculate 400 mL culture in LB + appropriate antibiotic and grow 18–24 h

17. Purify DNA via alkaline lysis. We use a nucleobond BAC 100 Maxiprep kit from Takara.

***Alternatives:*** The kit that has worked the best in our hands is the Nucleobond BAC 100 kit (Takara) but it is a fairly standard large-scale alkaline lysis column purification. Any other such kit designed for working with low-copy plasmids should work and kit-free protocols for efficient isolation of BAC DNA are also suitable (Villalobos et al., 2004).

18. Quantitate DNA concentration.

***Note:*** Nick translation theoretically only requires DNA at 65 μg/mL but a more concentrated stock nearly always works better. A range between 100 and 200 μg/mL is sufficient in our hands. (troubleshooting 4)

19. Pre-make reagents for nick translation
  a. Prepare nick translation buffer
  b. Dilute β-mercaptoethanol
  c. Mix nucleotide stocks

***Note:*** These reagents are kept at −20°C for long term storage. We do however recommend aliquoting nucleotide stocks to avoid freeze-thaw cycles and using opaque tubes to protect nucleotide stocks from light.

20. Prepare hybridization buffer
  a. Combine all ingredients
  b. Mix by gentle rotation for at least 18 h at 20°C–22°C
21. Make SSC dilutions

***Note:*** Hybridization buffer and SSC at various dilutions can be kept at 20°C–22°C for long periods of time. All other buffers should be made fresh.

### Optimization of nick translation with new DNAse

**Timing: 4 h, including a 2 h incubation**

***Note:*** This step is necessary whenever opening a newly purchased and prepared DNAse stock (not aliquot). It is also best practice, when beginning optimizing for a new probe, especially if you have one or a few probes that you plan on using for multiple experiments. It is a timecourse and a titration experiment to determine optimal DNAse concentrations and reaction times. The specific concentrations and times listed are suggestions only.

**Figure 2:**
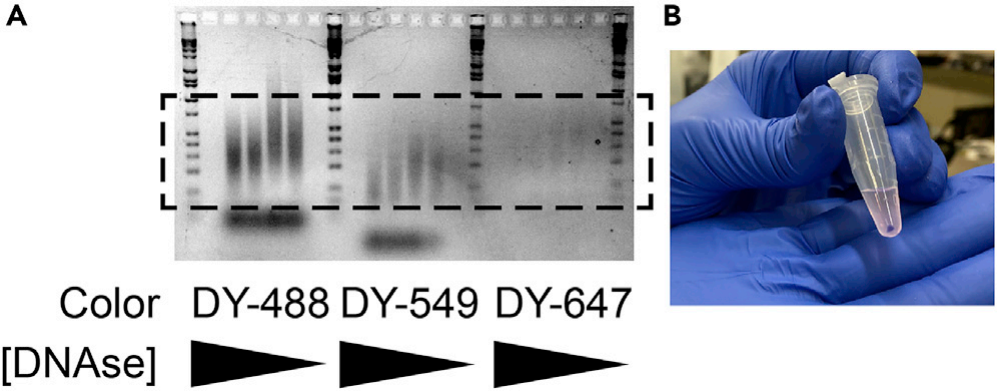
Quality control for probe preparation (A) Probes run on a 2% agarose gel counterstained with GelRed. The image shows probe lengths for three colors of tagged dUTP; note that different types of dUTP will integrate more or less efficiently and result in smears of different lengths and brightnesses. Decreasing concentration of DNAse from left to right within a color. Optimal probe length between 100bp and 1000bp marked with dashed lines. (B) Brightly colored, precipitated probe pellet.

22. Mix five working solutions of DNAse by diluting PCR-grade water:
  a. 1:2000
  b. 1:1000
  c. 1:500
  d. 1:200
  e. 1:100
23. Prepare five nick translation reactions (as described below, ideally with prepared BAC DNA that has been successfully used for FISH before) with each DNAse working solution above.
24. Incubate one of each working solutions from step 22) at 16°C for each of the following timepoints:
  a. 45 min
  b. 60 min
  c. 75 min
  d. 90 min
  e. 120 min
25. Stop reactions by addition of EDTA and heat treatment as described below. Once reaction has been stopped, keep on ice or at −20°C until all are finished.
26. Run all products on a gel as described below to determine optimal baseline concentrations and cutting times (Figure 2).

## Key resources table

**Table:** 

REAGENT or RESOURCE	SOURCE	IDENTIFIER
**Chemicals, peptides, and recombinant proteins**		

1M Tris HCl, pH 8.0	Thermo Fisher	Cat#15568025
1M Tris HCl, pH 7.5	Thermo Fisher	Cat#15567027
1M MgCl_2_	Quality Biological	Cat#351-033-721
BSA, lyophilized powder	Millipore Sigma	Cat#A9418
100 mM dATP	Thermo Fisher	Cat#10216018
100 mM dGTP	Thermo Fisher	Cat#10218014
100 mM dCTP	Thermo Fisher	Cat#10217016
1 mM DY488-dUTP (listed as fluorescent nucleotide stock within the protocol)	Dyomics	Cat#488-34
1 mM DY549P1-dUTP (listed as fluorescent nucleotide stock within the protocol)	Dyomics	Cat#549P1-34
1 mM DY647P1-dUTP (listed as fluorescent nucleotide stock within the protocol)	Dyomics	Cat#647P1-34
β-Mercaptoethanol	Bio-Rad	Cat#1610710
DNAse I grade II, lyophilized powder	Roche	Cat#11284932001
*E. Coli* DNA Polymerase I	NEB	Cat#M0209S
3M Sodium Acetate (NaOAc)	Thermo Fisher	Cat#R1181
0.5M EDTA	Thermo Fisher	Cat#15575020
1 mg/mL Human Cot-1 DNA	Thermo Fisher	Cat#15279011
10 mg/mL Yeast tRNA	Thermo Fisher	Cat#AM7119
50% w/v Dextran sulfate	Millipore Sigma	Cat#S4030
Deionized formamide, pH 7.0	Ambion	Cat#AM9344
20× Saline sodium citrate (SSC)	Corning	Cat#46-020-CM
10% Tween-20	Millipore Sigma	Cat#P9416
16% Paraformaldehyde (PFA)	Electron Microscopy Sciences	Cat#15710
10% Triton X-100	Millipore Sigma	Cat#X100
Saponin from Quillaja bark, powder	Millipore Sigma	Cat#S7900
1N HCl	Fisher Scientific	Cat#SA48
4′6-Diamidino-2-phenylindole (DAPI)	Millipore Sigma	Cat#9542
Glycerol	Millipore Sigma	Cat#G5516
100% Ethanol	Millipore Sigma	Cat#E7023
Phosphate Buffered Saline (PBS)	Millipore Sigma	Cat#D8537

**Critical commercial assays**		

Nucleobond BAC 100 Maxiprep	Takara	Cat#740579

**Deposited data**		

DNA FISH raw images	4D Nucleome	4DNFI2VH2VA2
FISH Spot positions	4D Nucleome	4DNFID78GRES
SpotLearn pipeline	GitHub	https://github.com/CBIIT/Misteli-Lab-CCR-NCI/tree/master/Gudla_CSH_2017

**Experimental models: cell lines**		

HFF c6 hTERT immortalized human foreskin fibroblasts	4DN	4DNINE5V67ON

**Other**		

1.5 mL DNA LoBind Tube	Eppendorf	Cat#022431021
1.5 mL LightSafe microcentrifuge tube	Millipore Sigma	Cat#Z688312
CellCarrier-384 Ultra Microplates	PerkinElmer	Cat#6007550
Aluminum foil plate seal	Greiner	Cat#676090
Water bath set to 16°C	N/A	N/A
Water bath set to 37°C–45°C	N/A	N/A
Benchtop microcentrifuge for 1.5–2.0 mL tubes	N/A	N/A
Vortex	N/A	N/A
Cooled microcentrifuge for 1.5–2.0 mL tubes	N/A	N/A
Tabletop plate centrifuge	N/A	N/A
Humidified incubator for cell culture	N/A	N/A
Humidified incubator for hybridization	Fisher Scientific	15-015-2632
Utility sponge (optional)	N/A	N/A
Slide moat (preferred) or heat block	Daigger	EF14654
Thermoshaker (preferred) or heat block	N/A	N/A
500 nm Tetraspeck fluorescent beads	Thermo Fisher	Cat#T281

***Alternatives:*** We list suppliers for standard molecular biological buffers and reagents. In most cases similar products from other suppliers can be substituted with no issue.

***Alternatives:*** This protocol has been optimized for use on plastic 384-well imaging plates, and we did not observe any difference between plates from various vendors. However, glass-bottom plates frequently do not stand up to the denaturation step, as glass and plastic have different thermal expansion coefficients and this results in cracks and leaks. This protocol could be scaled up for use in 96-well plates, but the required volume of hybridization mix would make those experiments expensive. We have, however, used a similar technique effectively on coverslips, adapting it only by performing the hybridization step on a slide sealed with rubber cement and performing washes by moving coverslips between wells of a 24-well dish.

## Materials and equipment

**Table:** 

10× nick translation buffer	Final concentration	Amount
1 M Tris-HCl, pH 8.0	0.5 M	500 μL
1 M MgCl_2_	50 mM	50 μL
10 mg/mL BSA	0.5 mg/mL	50 μL
ddH_2_O	n/a	400 μL
**Total**	**n/a**	**1 mL**

**Table:** 

0.01 M β-mercaptoethanol	Final concentration	Amount
β-mercaptoethanol	10 μM	0.7 μL
ddH_2_O	n/a	999.3 μL
**Total**	**n/a**	**1 mL**

Mix both nick translation buffer and β-mercaptoethanol dilution by vortexing, and store at −20°C for up to 6 months.

**CRITICAL:** β-mercaptoethanol is very pungent and can be irritating upon inhalation. Working with it in a fume hood is critical for the health of the experimenter.

**Table:** 

Nucleotide mixture stock	Final concentration	Amount
25 mM dATP	166 μM	4 μL
25 mM dCTP	166 μM	4 μL
25 mM dGTP	166 μM	4 μL
1 mM Dyomics labeled dUTP	166 μM	100 μL
1 M Tris pH 7.4	20 mM	8 μL
ddH_2_O	n/a	280 μL
**Total**	**n/a**	**400 μL**

Make on ice, mix by vortexing. Aliquot to prevent freeze-thaw cycles, store in opaque tubes to protect from light at −20°C. Store for up to 6 months.

***Alternatives:*** We use Dyomics directly labeled dUTPs as we find their dyes to be both bright and stable. We have also used Alexa labeled dUTPs from Thermo Fisher, Cy-dye labeled dUTPs, and dUTPs conjugated to a hapten such as biotin or digoxygenin which can be resolved in a secondary step, all with good results.

**Table:** 

Hybridization buffer	Final concentration	Amount
50% w/v dextran sulfate	15%	300 μL
Deionized formamide, pH 7.0	50%	500 μL
20× SSC	2×	100 μL
10% Tween-20	1%	100 μL
**Total**	**n/a**	**1 mL**

Mix by gentle inversion for at least 18 h to thoroughly mix without adding bubbles. Store at 20°C–22°C. Store for up to 6 months.

**CRITICAL:** Formamide is a hazardous substance and a known teratogen. It must be disposed of properly, and working with it in a fume hood is critical for the health of the experimenter.

**Table:** 

Permeabilization buffer	Final concentration	Amount
10% Triton-X 100	0.5%	500 μL
Saponin	0.5% w/v	50 μg
PBS	n/a	QS to 10 mL
**Total**	**n/a**	**10 mL**

Mix by vortexing. Make fresh on the day of the experiment.

**Table:** 

Equilibration buffer	Final concentration	Amount
20× SSC	2×	1 mL
Formamide	50%	5 mL
ddH_2_O	n/a	4 mL
**Total**	**n/a**	**10 mL**

Mix by inversion. Make fresh on the day of the experiment.

**CRITICAL:** Formamide is a hazardous substance and a known teratogen. It must be disposed of properly and working with it in a fume hood is critical for the health of the experimenter.

**Table:** Other solutions:

Name	Reagents
DNAse 1 stock	1 mg/mL DNAse in 50% glycerol. Store at −20°C indefinitely.
0.1N HCl	1N HCl, ddH_2_O. Make fresh.
DAPI stock	5 mg/mL DAPI in PBS. Store at 4°C for up to six months.
10 mg/mL BSA	10 mg/mL BSA in ddH_2_O. Store at −20°C indefinitely but avoid freeze-thaw cycles.

This protocol is designed to prepare cells grown on plates to be imaged in a high-content screening microscope such as the Perkin Elmer Opera or Yokogawa Cell Voyager systems. As FISH spots are generally diffraction limited, our best results have required a 60× water immersion lens (NA 1.2) and no binning, but deconvolved images detected with an epifluorescent scope at high magnification work similarly well as confocal images.

## Step-by-step method details

### Nick translation (sufficient for 3 wells of a 384-well plate)

**Timing: 4 h**

This step randomly digests a BAC probe into <1000 base pair linear segments, amplifies them by duplicating them with polymerase, and directly labels these segments through the addition of fluorescently tagged dUTP.

The volumes described below, for 1 μg starting probe DNA in a total reaction volume of 25 μL, are generally suitable to make enough probe to stain 3 wells of a 384-well plate.

In our hands, nick translation reactions have been successfully scaled up from a total volume of 25 μL as described here to a maximum of 500 μL. We do not recommend scaling up an individual reaction farther than this. If more than 500 μL of probe is required, set up several reactions up in parallel, and then pool the concentrated products.

1. Mix the nick translation reaction, on ice and protected from light.
  a. Dilute DNAse in PCR-grade water 1:1000 to a final concentration of 1 μg/mL
    **CRITICAL:** Retest this dilution with known probes every time you open a fresh 1 mg/mL stock of DNAse, as the appropriate concentration varies between 1:500 and 1:2000.
    ***Note:*** Depending on the probe used, this dilution can be adapted from 1:500–1:2000.
  b. Dilute DNA to 65 μg/mL in PCR-grade water on ice by mixing:
    i. 1 μg probe DNA
    ii. Fill to 15.5 μL PCR-grade water
  c. Mix master mix (scaled up as necessary) on ice and protected from light:
    i. 2.5 μL 10× nick translation buffer
    ii. 2.5 μL fluorescent nucleotide stock
    iii. 2.5 μL β-mercaptoethanol
    iv. 1 μL diluted DNAse stock
    v. 1 μL DNA polymerase
  d. Add 9.5 μL master mix per 2 μg probe DNA to each reaction tube
  e. Tap tubes to remove bubbles and spin 1 min at 20,000 *g* in a desktop centrifuge.

2. Incubate at 16°C in a water bath in a cold room for 80 min

***Note:*** we have tried anywhere from 30 min to 3 hours with varied results. Retest this when you are troubleshooting a new probe (troubleshooting 5).

3. Stop the reaction:
  a. Add 1 μL of 0.5 M EDTA
  b. Incubate at 72°C for 10 min
4. As a quality control step, run 5 μL of the reaction volume on a 2% agarose gel at 60 V for 30 min. Verify that probes run as a smear between 100 bp and 1000 bp (troubleshooting 5) (Figure 2A).

**Pause point:** Nick translated probes can be stored at −20°C for up to six months, although freeze-thaw cycles should be avoided. We have had the best results with relatively freshly made probes, stored for up to a week.

### Probe precipitation and resuspension in hybridization mix (sufficient for 3 wells of a 384-well plate)

**Timing: 4 h to 1 day**

Probes generated by nick translation are diluted in an aqueous solution and must be resuspended in hybridization mix for the FISH protocol. They also still contain residual DNAse and DNA polymerase. We use an ethanol precipitation step to clean the DNA and concentrate it, which allows us to resuspend it in hybridization mix.

The volumes described here, starting with 20 μL of nick translated probe in each of three colors, are usually suitable for staining 3 wells of a 384-well plate.

5. Mix the following in a micro-centrifuge tube:
  a. 20 μL nick translated probe in each color you will be using
  b. 9 μL human Cot-1 DNA (competitor; final concentration: 25 ng/μL)
  c. 6 μL yeast tRNA (carrier; final concentration: 165 ng/μL)
  d. 12 μL NaOAc
  e. 150 μL ethanol
6. Vortex to mix and spin 1 min at 20,000 *g* in a desktop centrifuge
7. Chill at −20°C for 60 min
8. Spin at 4°C at 20,000 *g* for 30 min
9. Gently decant supernatant and air dry tubes while inverted and protected from light for 10 min

***Note:*** The pellet at this point should be brightly colored (Figure 2B). Its color depends on the fluorophores used. Green fluorophores (such as DY-488) tend to make pellets more yellow, red fluorophores (such as DY-549) tend to make pellets more pink, and dark red fluorophores (such as DY-647) tend to make pellets more blue. (troubleshooting 6)

***Note:*** To remove residual ethanol without over-drying the pellet, we often use a shorter drying step and dry the sides of the tube with a Kimwipe^TM^ (Methods video S1).

10. Resuspend the pellet
  a. Add 45 μL hybridization buffer
  b. Heat to 65°C and vortex, or use a thermomixer at 65°C and 700+ rpm for at least 60 min (troubleshooting 7)

**Pause point:** Resuspended probes in hybridization mix can be stored at −20°C for up to six months, although freeze-thaw cycles should be avoided. We have had the best results with relatively freshly made probes, stored for up to a week.

Methods video S1. Use of a KimwipeTM to remove residual ethanol after precipitation of probes, related to step 9

### Fixation, washes, and permeabilization of the plate

**Timing: 1 day**

This step prepares cells for the hybridization process. Cells are fixed with paraformaldehyde, permeabilized to ensure access of probes through cell and nuclear membranes, chromatin is slightly deproteinated to allow probes to bind, and finally samples are equilibrated in a formamide solution to destabilize the DNA base pairing and allow denaturation to occur at lower temperatures.

For all washes in this step, we use 100 μL per well of a 384-well plate, but high precision is not necessary. For techniques to exchange media in 384-well plates, see Methods video S2.

11. Fix cells directly from cell culture
  a. Add 50 μL 8% PFA in PBS to wells already containing cells and 50 μL media (final: 100 μL of 4% PFA in PBS).
  b. Incubate 10 min at 20°C–22°C
  c. Rinse thrice in PBS

***Note:*** 8% PFA in PBS is best made fresh the day of the experiment, but can be stored for up to a week at 4°C.

**CRITICAL:** Formaldehyde is a hazardous substance. It must be disposed of properly and working with it in a fume hood is critical for the health of the experimenter.

**Pause point:** Cells can be held here up to 24 hours at 4°C in PBS or transferred to 70% ethanol and kept at −20°C for up to a week. Longer than that and signals start degrading, especially if using oligo-library based probes or plasmid probes. At this step, plates should be sealed with parafilm and/or plastic plate seals to prevent drying out during storage. If cells are stored after fixation, bring them fully to 20°C–22°C before continuing.

12. Incubate in permeabilization solution for 20 min at 20°C–22°C.
13. Rinse twice in PBS
14. Denature in 0.1N HCl for 15 min at 20°C–22°C
15. Neutralize in 2× SSC for 5 min at 20°C–22°C
16. Incubate in equilibration buffer for at least 30 min at 20°C–22°C.

**Pause point:** Cells in equilibration buffer can be kept at 4°C for a few days or up to a week. Longer incubations in fact often improve staining of larger regions. At this step, plates should be sealed with parafilm and/or plastic plate seals to prevent drying out during storage.

Methods video S2. Techniques of performing exchange of washes in plates: shaking over a reservoir and gentle extraction with a sponge, related to steps 11–16, 23–27, and troubleshooting 2 and 3

### Hybridization and washes

**Timing: 2–4 days**

This step combines the probe with the cells, allows stable binding, and washes off excess probe before imaging.

For all washes in this step, we use 100 μL per well of a 384-well plate, but precision is not necessary.

17. Add probe mix to wells
  a. Aspirate equilibration buffer gently with a 200 μL micropipette
  b. Immediately add 13.5 μL probe mix (Methods video S3)

**CRITICAL:** Do not let cells get dry at this step. We often go 3 wells at a time, pipetting probe mixes by hand. Use of an automated liquid handler that can handle viscous solutions (as the hybridization solution is quite viscous) may facilitate this step.

**CRITICAL:** Do not fully empty pipette when adding probe mix; this will cause bubbles that can interfere with the experiment.

18. Seal plate with foil plate seal to block light and prevent evaporation
19. Tap plate firmly against benchtop to disrupt bubbles (Methods video S3)

**CRITICAL:** It is recommended to add fiducial beads to two wells on the plate at this step, as using beads to check proper alignment between imaging channels and realigning images in post-processing is recommended for high precision measurements. However, without cells to stick to beads tend to float in PBS, and so beads should be dried onto an empty well. Thus, if you are using beads as fiducial markers, do not cover these wells with foil.

20. Spin plate 1 min at at least 100 *g* in a plate centrifuge
21. Denature by placing plate directly onto preheated slide moat, at 85°C for 7.5 min

***Note:*** Most slide moats do not have completely flat surfaces; make sure that all wells are fully in contact with the metal surface of the slide moat.

***Note:*** This hybridization temperature and time has worked well for us for a variety of cell and probe types, but during optimization with different cell types and probe types we have tested anywhere from 75°C to 95°C and anywhere from 3 to 10 minutes (see troubleshooting 8).

22. Immediately move to humid hybridization chamber at 37°C, hybridize at least 18 h

***Note:*** Hybridization of repetitive regions is typically completed in around 30 minutes, and unique genome regions should be largely hybridized within 18 hours of hybridization.

23. Rinse once in 2× SSC at 20°C–22°C

***Note:*** This step mostly just dissolves and lifts the hybridization solution. You can instead aspirate the hybridization solution with a pipette if cells are sufficiently adherent.

24. Wash three times for 5 min in 1× SSC at 42°C
25. Wash three times for 5 min in 0.1× SSC at 42°C

***Note:*** These wash conditions have worked well for us with a variety of cell and probe types, but during troubleshooting we have tested a wide variety of different wash conditions including: changing the concentration, duration, and temperature of these washes, as well as adding formamide to these wash steps (see troubleshooting 9, troubleshooting 10).

26. Stain with DAPI
  a. Dilute DAPI in PBS
  b. Incubate 10 min at 20°C–22°C
27. Rinse once with PBS
28. Plate with PBS, seal plate with foil

***Note:*** Imaging of high-throughput experiments in 384-well format requires an automated microscope designed for high content imaging such as a Perkin Elmer Opera, Yokogawa CV8000 or similar.

**Pause point:** Stained cells can be kept at 4°C, sealed as described, for up to one month before imaging, although prompt imaging is recommended.

Methods video S3. Technique of applying hybridization mix onto plates while minimizing bubbles formed, related to steps 17–19

### Image acquisition

**Timing: 3–12 h**

Routinely, we image 10–20 fields per well, in four channels, in a z-stack around 10 microns high and with slices every half micron, in each of 45–100 wells per experiment.

29. If stored at 4°C prior to imaging, warm plate at 20°C–22°C for one hour or at 37°C for 15 min to prevent condensation on the bottom of the plate.
30. Meanwhile, turn on the water line on the microscope for water immersion lenses
31. Clean the bottom of the plate with 70% ethanol and load it into the microscope
32. Find the approximate focal plane and height of the cells in the DAPI channel
33. Verify the signal-to-noise in all channels used: DAPI should be at least 5:1 and FISH signals should be easily discernible.
34. Program in the following image acquisition steps. Each channel’s acquisition should be unbinned and with the same z-stack, centered around the determined software focus. Optimize the laser power and exposure time so that all FISH channels have good signal-to-noise ratios (3-fold is ideal) and the signal is not oversaturated:
  a. Software focus optimizing the DAPI channel focus within a 10 μm z-stack surrounding the approximate focal plane found in step 32.
  b. DAPI image acquisition (Ex: 405, Em: 445/50)
  c. Dyomics DY488 or another green fluorophore acquisition (Ex: 488, Em: 525/50)
  d. Dyomics DY549P1 or another red fluorophore acquisition (Ex: 561, Em: 600/27)
  e. Dyomics DY647P1 or another deep red fluorophore acquisition (Ex: 640, Em: 676/29)

***Note:*** When selecting your z-slicing interval, consider whether you will be analyzing data as maximal projections (i.e. in 2D) or 3D volumes. If the former, a z-slice interval around the same size as the depth of field of the lens (around 1 μm) will limit imaging time while not missing any signals. However, in 3D this often causes artifacts as voxels are extremely uneven in size and 3D centers cannot be determined with great precision. As such, for 3D analyses we recommend oversampling in z by at least 3-fold (i.e. a z-slice interval around 300 nm).

**CRITICAL:** Do not optimize imaging conditions in only one well if different probes are used in different wells as there can be considerable variability across the plate. We recommend verifying imaging conditions in at least 5–10 wells.

35. Program the microscope to acquire sufficient fields of view to image 1,000 cells per condition.
36. Save and launch the routine.

## Expected outcomes

A good FISH result will have several features. Because of the high-throughput nature of this pipeline, in which a single experiment can easily result in over 1000 images per channel, visual inspection of every image is not feasible. But we strongly recommend visual inspection of a subset of the images to complement systematic quantification for quality control.

Overgrown cells frequently become senescent, and cells that are too sparse frequently exhibit a stress response, both of which can alter results. Furthermore, from a purely technical perspective, cells that are too sparse will require much more time to image (Figure 1A), and cells that are too dense frequently overlap and can confound segmentation algorithms (Figure 1B). **Ideal density is generally around 80% confluency for adherent cells** (Figure 1C). (troubleshooting 3)

Probe size and quality is probably the most important determinant of a DNA FISH experiment. We recommend running probes on a gel to determine their size. Expect probes to be between 100 bp and several thousand bp in length (Figure 2A). Precipitated probes should furthermore be brightly colored, and this can serve as a measure of the efficiency at which fluorescent nucleotides have been incorporated into the probe (Figure 2B). (troubleshooting 5, troubleshooting 6)

The DAPI channel typically serves to identify individual cells during image analysis but can also be used to diagnose issues with permeabilization. Over-permeabilized cells will often show disruptions of the typical chromatin staining pattern in the DAPI channel (troubleshooting 8.)

**Figure 3:**
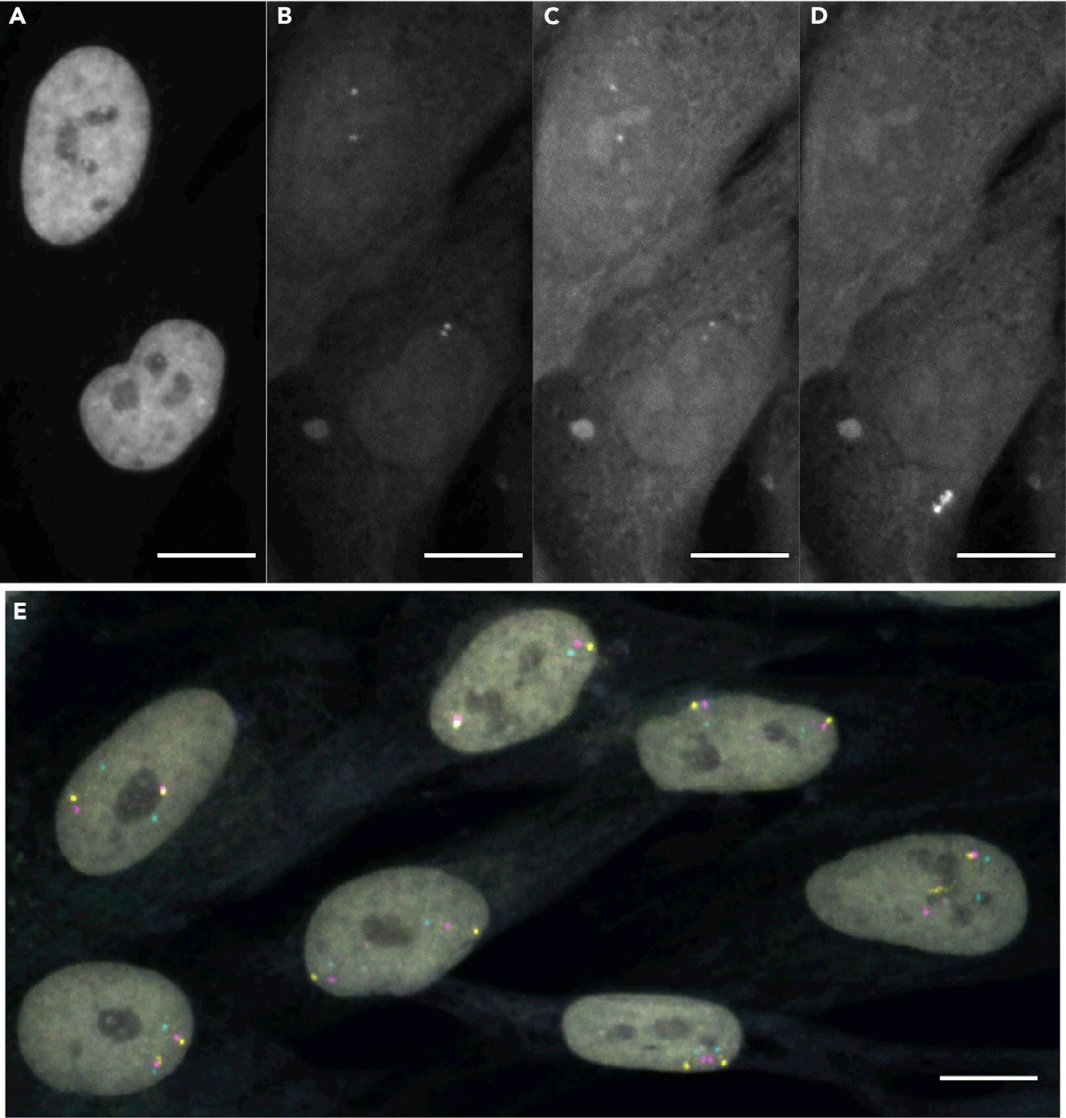
Signal-to-background ratio as a metric for a successful FISH (A) DAPI staining showing position of nuclei for following three images. (B) Relatively faint signals are easily distinguished with low background. (C) High background makes segmenting signals harder and nucleolar background or structure within the background can lead to mis-segmented spots. (D) Very low signal becomes entirely indistinguishable from background. (E) Exemplar FISH with high signal to background ratios in all three channels. Scale bars: 10 microns.

A “good” FISH signal is one that is easily discernible above nuclear background. We have observed that almost all segmentation pipelines – including proprietary commercial systems such as PerkinElmer’s Acapella or Columbus (Finn et al., 2017), lower-throughput hands-on methods such as ImageJ macros, and open source deep-learning based platforms such as SpotLearn (Gudla et al., 2017) -- perform well with FISH signals ∼3-fold higher than nuclear background (Figure 3B). FISH signals can be difficult to properly identify in an automated pipeline if the signal is overall very faint (Figure 3C) or if the background is generally high, especially in the nucleus (Figure 3D). Checking your signal to noise ratio in a handful of images after imaging has completed and/or at the time of imaging is recommended. Little can be done in post-processing to save a FISH with a low signal to noise ratio. (troubleshooting 9, troubleshooting 10).

**Figure 4:**
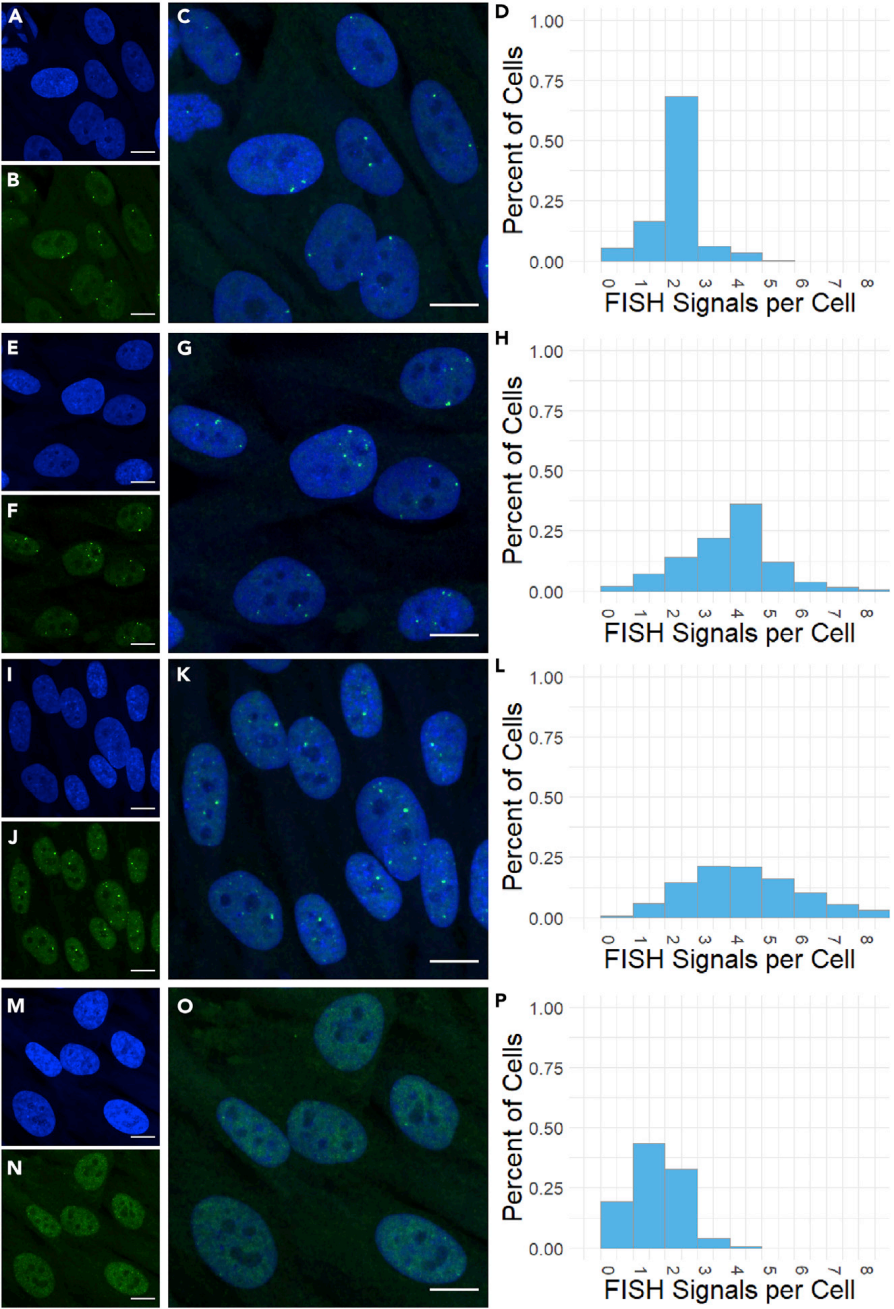
Spots per cell as a measure for detection efficiency (A) DAPI for nuclei of well-stained FISH to a diploid locus. (B) Successful FISH to a diploid locus. (C) Overlay of (A and B). (D) Histogram of per-cell spot segmentation for this experiment, showing most cells have two segmented spots. (E–H) As (A–D), but with a probe that may be duplicated or contaminated; most cells show four spots and there is more variability. (I–L) As (A–D), but with a probe that has spotty background and a very wide range of spots segmented per cell. (M–P) As above, but with a probe that has very faint staining and very few spots segmented per cell. All scale bars are 10 microns.

Based on the ploidy of the cells, the number of expected signals per cell is known: for normal diploid cells, most FISH probes should stain two spots per cell. This provides a way to measure your detection efficiency after automated segmentation has been done. Good probes to single-copy genes in diploid cells should detect two spots in most of the cells (Figures 4A–4D). Probes to repetitive regions or regions which have been duplicated and translocated within a cell line will result in multiple signals in most of the cells, with the number of signals depending on the type of genomic abnormality present in the cell (Figures 4E–4H). Probes that simply failed frequently result either in a wide range of total spots detected in a cell (Figures 4I–4L) or very few spots segmented in any cell (Figures 4M–4P). (troubleshooting 11)

Because of random drift, uncorrected aberrations, and misalignment between the multiple cameras in the instrument, all microscopes have a distance threshold below which they cannot accurately detect co-localization or measure distances. **Therefore, it is critical to determine the resolution limit of your microscope with your setting experimentally, and to include these controls in every experiment.**

**Figure 5:**
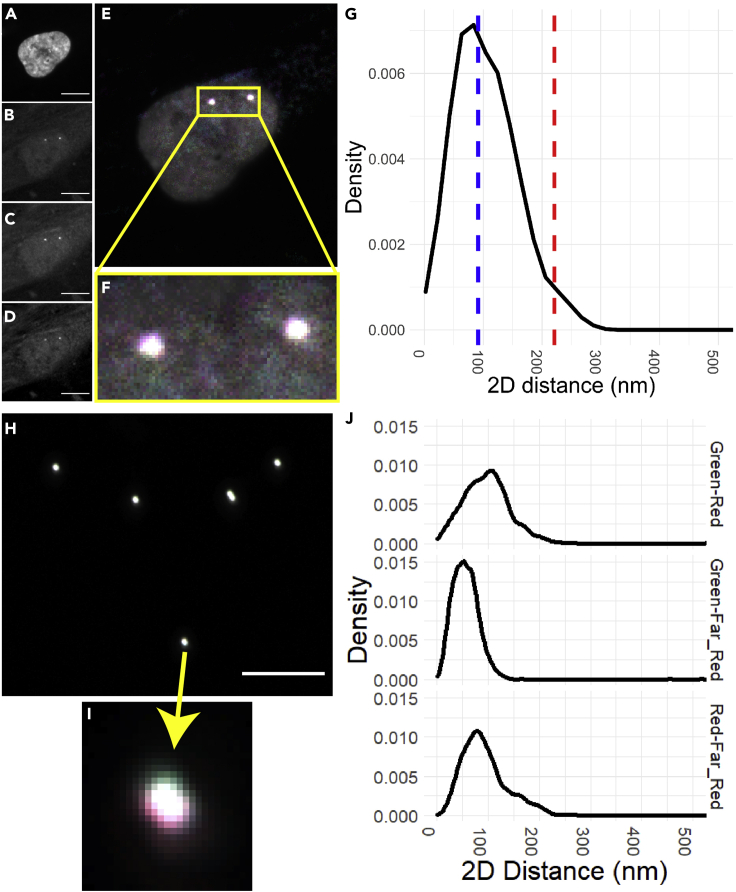
Costained controls and beads to measure misalignment (A–E) Representative image of a FISH experiment with one locus stained in two colors. (A) DAPI, (B) DY488, (C) DY549P1, (D) DY647P1, (E) Composite. Scale bar is 10 microns. (F) inset close-up of two spots to see overlap. (G) Distance distribution of all control spot pairs. Median (blue dashed line) is 97nm and 95^th^ percentile (red dashed line) is 221nm. (H) Representative image of fiducial beads in all four colors (Tetraspeck^TM^ 500 nm beads, Invitrogen). Scale bar is 10 microns. (I) inset close-up of one bead to see overlap. (J) Distance distributions of all beads, post-correction, showing each color combination. Note that misalignment varies by color pair.

Determining the resolution limit experimentally ensures you do not run afoul of a detection limit when trying to measure distances. We apply two approaches: beads and a co-stained locus. Our preferred control is a single region, stained in all relevant colors (Figures 5A–5F) which is integrated into the experiment and provide markers to validate alignment on a per-experiment basis, controlling for misalignment in the microscope as well as any aberrations generated by non-uniform diffraction through the cell (Figure 5G). Fiducial beads can also be dried onto the plate during the hybridization step (see above; Figures 5H and 5I) and provide markers to validate microscope alignment on a per-experiment level (Figure 5J).

## Quantification and statistical analysis

### Image processing techniques

Proper image post-processing, segmentation, and automated analysis is a major challenge in working with large imaging datasets. Each new dataset and experiment will require some customization in its analysis. We outline below several general principles to consider in analyzing large imaging datasets, with a specific focus on measuring 2D distances between DNA loci in DNA FISH images. A more detailed description of one pipeline we have used has been previously published (Gudla et al., 2017).

Our image processing pipeline generally includes the following steps:

1. Calculate maximum projections of each field
2. Segment nuclei in the maximum projection of the DAPI channel
3. Segment all FISH spots within nuclei
4. Calculate center of gravity for each spot
5. Measure all distances between spots of different colors within a cell
6. Select minimum distances on a per-spot per-channel-pair basis (i.e., minimum distance between red and green spots on a per green spot basis)

Note that confocal images contain many other measurable parameters than 2D position. Calculating parameters such as 3D position, radial position, domain size or overlap for large regions (full area half max), and integrated intensity in any channel are straightforward analyses to add onto a pipeline such as the one described above. While we largely focus on spot-to-spot distances, simplifying imaging datasets into distance measurements loses much of the richness of the images themselves.

Since these steps are performed on each image, our pipelines have significantly benefitted from parallel processing. While analyzing each image in parallel is perhaps simplest, cropping out segmented nuclei and processing instead each nucleus in parallel has also given us noticeable performance gains. However, this is most significant on datasets of thousands of images, and smaller datasets can be analyzed readily without any parallel processing.

Machine learning has also increased the efficiency and quality of image processing pipelines. In particular, segmentation of nuclei that are almost touching or slightly overlapping is significantly improved using machine learning-based tools such as CellPose (Stringer et al., 2021). While machine learning can also be applied to spot segmentation (Gudla et al., 2017), we have observed that the benefit here is not as great and in general it is better to improve the quality of the FISH experiment until the signal to noise ratio is sufficient for easy segmentation using traditional methods.

### Quantitative quality control

Whereas in traditional imaging with relatively small datasets, examination and quality control of each image by a trained scientist is feasible, when imaging datasets contain thousands or hundreds of thousands of images this is no longer possible. As such, since no human will actually look at all of the images analyzed, rigorous quality control based on quantitative parameters is necessary. We take several steps for this:

7. Examining one randomly selected field in each well visually, as a baseline.
8. Calculating the number of spots segmented per cell on a per-well basis, and using only those wells in which a majority of cells show two spots for a diploid locus. This corresponds roughly to a 70% per-chromosome detection efficiency (Figure 5).
9. Considering only cells in which the expected number of spots has been segmented in both relevant channels.
10. Considering only probes which are reliably found within the neighborhood (∼ 4 um) of other probes on the same chromosome. Probes which consistently stain regions very far from others supposedly on the same chromosome are likely to be mis-mapped or translocated.

### Statistical concerns

Special attention must be paid to statistical tests performed on distance distributions generated from large imaging datasets for two reasons: first, because distance distributions are not normal distributions, making certain common statistical tests less accurate; second, because datasets are very large, statistically significant results can be obtained for very small differences.

Distance distributions calculated from FISH images visualizing two genes invariably show positive skew, as is common with biological phenomena with minima of zero (Limpert and Stahel, 2011). They are not expected to follow the same rules as a normal or gaussian distribution. As such, nonparametric tests such as a Wilcoxon Rank Sum test are preferred to a T-test for measuring differences in the center of the distribution, and a test such as the Kruskall-Wallis test rather than an F test is a better choice for measuring differences in the variance or spread of the distribution (Ott and Longnecker, 2015). Performing a log-transform on distances may also increase the accuracy of tests and modeling (Limpert and Stahel, 2011).

Many statistical tests are also able to distinguish very small differences as statistically significant on very large datasets. In our datasets, differences as small as 1% are frequently highly statistically significant. There are two approaches to presenting this data. The first is to always report effect size as well as statistical significance. The second is to use methods akin to bootstrapping to multiply subsample and calculate likelihoods (p-values) or confidence intervals.

## Limitations

We have found high-throughput FISH using nick translated probes and confocal microscopy, as described here, to be robust and broadly applicable. However, there are several specific situations where other techniques may be more appropriate.

Nick translated probes are not optimal for targeting either very large (on the order of millions of base pairs) or very small (less than a fosmid, about 50,000 base pairs) genomic regions. Although probes can be generated by PCR-amplifying out the small target region and cloning it into a plasmid for nick translation, it will often be easier and more straightforward to use oligo-library based probes (Mateo et al., 2019). Similarly, if you intend to target hundreds or thousands of regions, it is more straightforward to design an oligo library and synthesize in a pooled manner, rather than individually purifying DNA from hundreds or thousands of bacterial artificial chromosome probes (Beliveau et al., 2015). Finally, oligo library-based probes will also be preferred when base-pair level control over the edges of your probed region is required, and BAC or fosmid probes cannot be found. In any of those cases, the hybridization steps of this protocol can be adapted to use such probes, but additional troubleshooting will likely be necessary.

Furthermore, there are significant limitations built into diffraction-limited confocal microscopes, and while adapting super-resolution microscopy to high-throughput contexts is currently an area of active research (Mahecic et al., 2020), these techniques currently max out at hundreds of human cells rather than the millions of human cells imaged by our platform. We generally observe a resolution limit on the order of ∼100 nm in our images, and measuring distances between regions that are separated by less than 100 nm will thus require super-resolution imaging such as PALM (Platzer et al., 2020). While confocal microscopy can determine spot centers to sub-pixel accuracy, and while DNA FISH signals are not perfectly round/gaussian in shape, edge positions cannot be precisely determined from diffraction-limited imaging. Therefore, doing volumetric analyses or measuring edges and overlap between two regions requires super-resolution imaging (Wang et al., 2016).

Finally, microfluidic approaches with multiple rounds of stripping and staining many regions in series (Chen et al., 2015) allow for much more combinatorial power to measure distances between many different probes all in the same cell. While testing hundreds of probe pairs is not unreasonable with the approach described here, each cell imaged with this approach only has three regions resolved. Doing analyses of large multi-gene clusters, or hundreds of regions all in the same cell, will require applying microfluidic approaches.

## Troubleshooting

### Problem 1

Suggestions for working with different types of probes. (Relevant to Before You Begin)

### Potential solution

We have adapted this protocol to stain large contiguous regions, as well as to decrease probe size for fosmid probes (∼50 kb) or cloned plasmid probes (∼10 kb), and oligo-library based probes. Here are some of the basic guidelines we have uncovered:

For directly labeled nick translated probes of varying lengths, the only thing we have had to change generally speaking is the final probe concentration in ng/μL. Probes staining longer regions need higher concentrations, and probes staining shorter regions require lower concentrations, although the difference is not as stark as might be suggested if trying to keep molarity roughly constant. When using a probe of a size very different than a BAC, we recommend a first experiment trying a few different concentrations at the resuspension step to optimize for signal-to-noise.

For nick translated probes with haptens that can be resolved with a secondary label, such as biotin- and digoxygenin-labeled probes, we performed the secondary labeling step after the SSC washes and before DAPI staining.

For oligo-library based probe mixes, obviously probe preparation will be entirely different and has been thoroughly described elsewhere (Beliveau et al., 2017). We have observed that these techniques are much more sensitive to the age of the cells, and using freshly fixed/permeabilized cells rather than storing for any length of time greatly improves efficiency. We have further seen improvements by amending the post-hybridization washes to three washes in 2× SSC at 42°C for 5 min and three washes in 2× SSC at 60°C for 5 min.

### Problem 2

Modifications to cell culture and treatment protocols are often necessary for different types of cells. (Relevant to Before You Begin.)

### Potential solution

We have tested this protocol on many different cell types with success. We have included a list of the modifications necessary for specific cell types below, to provide a starting point for your own troubleshooting.

PANC-1 cells, HBECs, and skin fibroblasts other than HFFs can be put through exactly the same protocol, unaltered.

HCT116 cells and other delicate adherent cells: Since these cells tend to be somewhat clumpy and detach more easily, we have the best results with them by plating at a higher density and performing washes with a sponge (Methods video S2).

H1 human embryonic stem cells and induced pluripotent stem cells: Several changes were useful, including plating at a lower density and growing in 384-well plates for at least two days in order to ensure colonies have time to flatten, rinsing the plate twice with PBS before fixation to remove autofluorescent cellular debris, and either plating for imaging as a monolayer of dissociated colonies (with a ROCK inhibitor to prevent spontaneous differentiation) or using a secondary control program such as Wako Automation Suite's Search First to identify colonies at a lower magnification and subsequently image them in a targetted fashion.

Suspension-grown cells or patient-derived cells which cannot be grown directly on the 384-well plate: these cells ought to be spun down onto the plate immediately before fixation, at very high density. You will lose many cells during the protocol as there are many wash steps. All washes must be done with a sponge, and we recommend doing fewer washes for longer times, increasing stringency by increasing temperature or decreasing wash concentration if necessary, as described in troubleshooting 9. To spin dissociated cells down onto a pre-warmed poly-D-lysine coated plate, we plate 50 μL of cell suspension containing 10^7^ cells/mL and spin at 250 *g* for 5 minutes immediately before fixing.

### Problem 3

Cells at time of imaging are too sparse, dense, or clumpy (Figure 1). (Relevant to before you begin steps 1–15)

### Potential solution

Doing a seeding density curve at the outset can help pinpoint an effective seeding density. However, there are several other possibilities as well, outlined below:

If the problem is limited to a few wells, and some wells are optimally confluent while others are spotty, try spinning your plate for 1 min at 250 *g* immediately after plating, which will remove bubbles which might get in between the cells and the bottom of the plate.

If the problem is cell clumping, and some fields are very full while others in the same well are sparse, let the plate rest for 30 min at 20°C–22°C before returning it to the incubator, to slow adhesion and allow cells to spread out.

Check to make sure your incubator is humid and the tray is full of water. Even partially dry wells do not grow well. If you have a humid incubator but your wells are still visibly drying out, try filling the rest of the plate with PBS or water.

Check in a dissecting scope after fixation, permeabilization, and during final wash steps to make sure you are not losing your cells during wash steps. If you are, use a utility sponge to remove liquids, as this is gentler. (Methods video S2)

### Problem 4

DNA yield too low for nick translation (Relevant to before you begin steps 16–18)

### Potential solution

We have observed three things that significantly help with improving DNA yields: first, growing bacterial cultures from a single colony each time, second, spinning the lysate after alkaline lysis to pellet the precipitate as well as filtering through a paper filter, and third, pre-warming elution buffer to 50°C before eluting DNA.

### Problem 5

Nick translation reaction does not yield appropriately-sized fragments. (Relevant to steps 1–4)

### Potential solution

If the problem is consistent across many BACs, consider replacing DNAse if your products are too long and replacing DNA polymerase if they are too short.

If the problem is limited to a few BACs, as different DNA sequences are differently sensitive to DNAse, consider doing a few titration experiments to optimize the nick translation protocol to those probes specifically, as you would when optimizing for a new aliquot of DNAse. Additional changes that may be fruitful include increasing the DNA concentration to 130 or 260 μg/mL and making fresh nucleotide mix stock solutions.

### Problem 6

Probe pellets are pale, faint, or tiny. (Relevant to steps 5–9)

### Potential solution

Sometimes this happens if insufficient ethanol is added to the precipitation. In other cases, it may be a problem with the nick translation reaction, or the fluorophores.

### Problem 7

Probes do not resuspend. (Relevant to step 10)

### Potential solution

Since hybridization mix contains formamide, probes should resuspend quickly and easily. Some white fluffy pellet remaining is normal but most of the probe and color should have gone into solution. But if they don’t, first consider shortening the drying time and using a Kimipe^TM^ to remove residual ethanol from the side of the tubes instead. (Methods video S1). If they still do not go into solution, pipetting repeatedly to mix with a 200 μL micropipetter will dislodge the pellets and break them apart to help them dissolve. Finally, DNA is very soluble in pure formamide. Making 2× formamide-free hybridization mix with all ingredients except the formamide, using half volumes of pure formamide to dissolve probes, and then adding the 2× hybridization mix may solve this problem for particularly difficult probes.

### Problem 8

Failed FISH combined with DAPI signal that is blurry or shows cytoplasmic background. (Relevant to steps 12 and 21)

### Potential solution

This can be caused by issues with permeabilization and denaturation. Over-permeabilization or denaturing cells too hot or too long can rupture the nuclear membrane and allow the DNA to leak out, disrupting the crisp nuclear edges resolved by DAPI. On its own, this will confound automated nuclear segmentation and cause problems downstream with the analysis, but this rupture also obviously changes the location of DNA in the cell and therefore even if the FISH appears to have worked the data is no longer trustworthy. The permeabilization steps can be made gentler by shortening the length of time in the Triton/Saponin solution to ten minutes, by decreasing the concentration of the Triton/Saponin solution to 0.01% each, by permeabilizing with ethanol instead of Triton/Saponin, by reducing the denaturation temperature to 75°C, or by reducing the denaturation time to 5 minutes.

### Problem 9

Signal is too faint, with low nuclear background. (Relevant to steps 1–4, 12, 21, 22, 24, and 25)

### Potential solution

There are several issues that can cause faint signals and low background. If you have already optimized the nick translation and are getting well-labeled and bright probes of an appropriate length, permeabilization or hybridization may be at fault. There are several possible causes of this, and each has its own possible solutions.

First, the cells could be insufficiently permeabilized. This can be resolved by lengthening the permeabilization time to 30 minutes, increasing the concentration of the Triton/Saponin solution to 1% each, or adding an enzymatic digestion step by incubating in pepsin.

Second, the genomic DNA could be insufficiently denatured. Solve this by lengthening the denaturation step to 10 minutes, increasing the temperature of the denaturation step to 95°C, or increasing the time of the equilibration step to overnight or longer.

Third, the hybridization time may be too short. Trying a longer hybridization step (such as 2-4 days) at a higher temperature (such as 42°C), will address this.

Fourth, the washes could be too stringent. Washing in higher concentrations of SSC, such as only washing in 2× SSC or 1× SSC, washing at lower temperatures from 20°C to 37°C, or removing wash steps post-hybridization entirely could solve this problem.

Replacing directly-labeled dUTP with dUTP conjugated to a hapten such as biotin or digoxygenin, and a secondary labeling step, will slightly amplify signals and may help. Note that this could also have effects on the shape and nanostructure of the DNA, and so is recommended as a last resort.

### Problem 10

Background is high and masks signal. (Relevant to steps 11–17, 21, 24 and 25)

### Potential solution

There are several issues that can cause high background in FISH. The most likely culprit is the probe, so the first step is to optimize the nick translation. If you have already optimized the nick translation and are getting well-labeled and bright probes of an appropriate length, fixation or hybridization may be at fault. Generally, the first step is to remake your hybridization solution. If this fails, consider where your background is coming from. High nuclear background with low cellular background is likelycaused by issues with the probe itself, while high cellular background is more likely caused by fixation or hybridization issues or native autofluorescence. In either case, there are experimental solutions.

For high cytoplasmic background, consider your cell type and fixation time. Overfixation frequently causes cytoplasmic background especially in the yellow-green range. Reducing the fixation time, removing cell culture media and rinsing with PBS prior to fixation, and reducing the PFA concentration to 1% will help. There are also many commercial systems for reducing autofluorescence in tissues. We have had limited success applying these to cultured cells, and suspect that it may be cell-type specific.

For high nuclear background and low cellular background, it is most likely a problem with the probe, and the first step would be to optmize the nick translation reaction as described in problem 5. However, for probes that have already been optimized, it could be a problem with the genomic DNA or the wash steps. To protect genomic DNA, use a freshly fixed plate, reduce permeabilization or acid treatment times or concentrations, or shorten the equilibration step prior to adding probes. To protect genomic DNA and probe DNA, reduce the denaturation temperature to 75°C or the denaturation time to 5 minutes. If none of these work, consider washing at higher temperatures (up to 65°C), for longer times (up to 15 minutes), or adding formamide to the washes (up to 50% formamide in 2× SSC.

### Problem 11

Bright signals with improper numbers based on karyotype for the cell line used. (Relevant to Before You Begin)

### Potential solution

This can be caused by a probe to repetitive/duplicated DNA (Figures 4I–4L). It could also be caused by cross-contamination during probe preparation. Often, we find that tracing the source of the issue is not worth the time, and the best thing to do is order a different BAC clone to the same, or an overlapping, genomic region.

## Resource availability

### Lead contact

Further information and requests for resources and reagents should be directed to and will be fulfilled by the lead contact, Tom Misteli (mistelit@mail.nih.gov).

### Materials availability

This study did not generate any new unique reagents.

### Data and code availability

The published article includes all datasets and code generated or analyzed during this study. Source image data is available via the 4D Nucleome project: 4DNFI2VH2VA2

The open source SpotLearn KNIME pipeline is available on GitHub at: https://github.com/CBIIT/Misteli-Lab-CCR-NCI/tree/master/Gudla_CSH_2017
